# Prescription drug coverage in Canada: a review of the economic, policy and political considerations for universal pharmacare

**DOI:** 10.1186/s40545-018-0154-x

**Published:** 2018-11-07

**Authors:** Jaden Brandt, Brenna Shearer, Steven G. Morgan

**Affiliations:** 1 College of Pharmacy, Rady Faculty of Health Sciences, University of Manitoba, Winnipeg, MB Canada; 2 Pharmacists Manitoba, Winnipeg, MB Canada; 3 School of Population and Public Health, Faculty of Medicine, University of British Columbia, Vancouver, BC Canada

**Keywords:** Pharmacare, Canada, Prescription drugs, Health insurance, Health policy, Healthcare economics, Politics

## Abstract

**Background:**

Canadians have long been proud of their universal health insurance system, which publicly funds the cost of physician visits and hospitalizations at the point of care. Prescription drugs however, have been subject to a patchwork of public and private coverage which is frequently inefficient and creates access barriers to necessary medicine for many Canadians.

**Methods:**

A narrative review was undertaken to understand the important economic, policy and political considerations regarding implementation of universal prescription drug access in Canada (pan-Canadian pharmacare). PubMed, SCOPUS and google scholar were searched for relevant citations. Citation trails were followed for additional information sources. Published books, public reports, press releases, policy papers, government webpages and other forms of gray literature were collected from iterative internet searches to provide a complete view of the current state on this topic.

**Main findings:**

Regarding health economics, all five of the reviewed pharmacare simulation models have shown reductions in annual prescription drug expenditure. However, differing policy and cost assumptions have resulted in a wide range of cost-saving estimates between models. In terms of policy, a single-payer, ‘first-dollar’ coverage model, using a minimum national formulary, is the model most frequently advocated by the academic community, healthcare professions and many public and patient groups. In contrast, a multi-payer, catastrophic ‘last-dollar’ coverage model, more similar to the current “patchwork” state of public and private coverage, is preferred by industry drug manufacturers and private health insurance companies. Primary concerns from the detractors of universal, single-payer, ‘first-dollar’ coverage are the financing required for its implementation and the access barriers that may be created for certain patient populations that are not majorly present in the current public-private payer mix.

**Conclusion:**

Canada patiently awaits to see how the issue of prescription drug coverage will be resolved through the work of the Advisory Council on the Implementation of National Pharmacare. The overarching and ongoing discourse on policy and program implementation may be construed as a political debate informed by divergent public and private interests.

**Electronic supplementary material:**

The online version of this article (10.1186/s40545-018-0154-x) contains supplementary material, which is available to authorized users.

## Background

Canadians have long been proud of their universal health insurance system, “Canadian Medicare” as it is affectionately known [1]. This system of public health insurance, coordinated between the provinces and the federal government through the *Canada Health Act*, keeps Canadians comforted in the knowledge that medically necessary physician visits, diagnostic tests and hospitalizations will be “taken care of” as a matter of course regardless of their age, income, or province of residence [2]. However, the benefits of Canada’s universal public health insurance system stop at the community pharmacy, where uninsured costs of medications result in financial hardship for many Canadians [3, 4]. This makes Canada unique insofar as it is the only high-income country with a universal health insurance system that does not provide universal coverage of prescription drugs.

Canada’s system of universal health insurance was not supposed to exclude prescription drugs. Canadian Medicare was a national health insurance system built in stages through conditional grants from the federal government to the provinces that are ultimately responsible for health care administration [5]. Since the outset of Canadian Medicare, national commissions have repeatedly recommended that universal public drug coverage – universal “Pharmacare” as that vision is often called in Canada – be part of Canada’s universal public health insurance system [6–9]. The topic of universal pharmacare has waxed and waned in past policy debates, often in accordance with the publication of reports by national commissions on Canada’s health care system [10]. Despite these brief windows of attention in the past, universal pharmacare has not been implemented in prior eras owing to various political and economic constraints [11]. Factors which have historically impeded progress on this front range from the large implementation cost that would be incurred by government, misalignment of views between policy actors at different levels and the historical lack of electoral incentives to make pharmacare an issue worthy of attention at the highest levels of political campaigning [11]. Nonetheless, recently pharmacare has become a central topic of national health policy debate and the gap between policy discourse and policy action appears to be narrowing as evidenced by the federal government’s creation of an Advisory Council on the Implementation of National Pharmacare in 2018 [12].

In this paper, we provide a narrative review and commentary on three major issues relevant to the current topic of universal pharmacare in Canada; the economics, policy options and political considerations of such a system. It is meant to provide an accessible summary of the complexities of Canada’s ongoing efforts to achieve a system of universal drug coverage. Beyond this, we believe it offers a compelling case-study (especially for those new to pharmaceutical policy) which details the multiple variables and factors that must be considered for policy decision making and program implementation at the highest levels of public health. For the reader wholly unfamiliar with the different entities operating in Canada’s pharmaceutical market environment, supplemental Additiional file 1 provides a brief overview that may be useful before proceeding into the following sections of the article.

## Methods

This narrative review was informed by a structured search strategy applied to PubMed, SCOPUS and google scholar using combinations of the terms: ‘pharmacare’, ‘Canada’, ‘Canadian’, ‘prescription drug’, ‘drug coverage’, ‘policy’, ‘formulary’, ‘provincial’, ‘national’. Citation trails were also followed from bibliographies to accumulate more information sources. Lastly, published books, public reports, press releases, policy papers, government webpages and other forms of gray literature were collected from iterative google searches to provide a complete view of the current state on this topic. There were no date restrictions, though preference was given where possible to newer information sources.

While the lack of a reproducible, systematic search strategy may be seen as a significant limitation of this review, we maintain that combining the various aspects of the pharmacare topic (economics, policy and politics) into a coherent work was more amenable to a realist, narrative strategy rather than a rigorous systematic approach. Lastly, all authors, having varying levels of professional experience relating to pharmaceutical policy in Canada were engaged in formulating the structure of the review to ensure its appropriate breadth and depth of content.

## Current prescription drug coverage in Canada

In contrast with its universal Medicare system, Canada’s system of prescription drug coverage involves a complex and largely uncoordinated mix of public and private insurance plans that differ in terms of eligibility, patient charges, and drugs covered (i.e., formularies) [13]. Though there are no national standards for public drug programs in Canada, each province offers some form of public subsidy for prescription drugs. These programs evolved beginning in the 1960s and 1970s, and traditionally provided relatively comprehensive public drug insurance for select population groups: specifically, persons on social assistance and persons over the age of 65 [14]. Today, the public drug plans offered by provinces vary more dramatically in terms of who is covered because some provinces – notably Manitoba and British Columbia – no longer provide comprehensive coverage for older residents. The greatest differences in public drug coverage in Canada are differences in drug benefits available for residents who are not on social assistance and age 65 or older. As summarized in Table 1, no province provides universal, comprehensive public coverage for this general population group.^1^ Instead, most provinces offer the general population coverage against “catastrophic costs” that exceed deductibles set as a percentage of household income. The percentages of household income used to define deductibles vary considerably across provinces that offer these catastrophic coverage programs. After a deductible limit is met, the government pays for all or a significant portion of the cost for eligible drug products.

**Table 1:** – Provincial Drug Coverage Programs for General Population (non-senior and non-social assistance)

	Patient Eligibility	Coverage Details
British Columbia	No restrictions	Catastrophic coverage with deductibles up to 3% of annual income, then patient pays 30% co-insurance.
Alberta	No restrictions	Voluntary coverage with premiums, then patient pays 30% co-insurance.
Saskatchewan	No restrictions	Catastrophic coverage with deductibles at 3.4% annual income, then patient pays 35% co-insurance.
Manitoba	No restrictions	Catastrophic coverage with deductibles up to 6.97% annual income, then full coverage of eligible drugs.
Ontario	No restrictions	Catastrophic coverage with deductibles at approximately 4% of annual income, then patient pays $2 fixed co-payment.
Quebec	Restricted to those not eligible for private insurance	Mandatory coverage with premiums and a $19.90 monthly deductible, then patient pays 34.9% co-insurance.
Newfoundland	No restrictions	Catastrophic coverage with out-of-pocket payments ranging from 5 to 10% of income
Nova Scotia	No restrictions	Catastrophic coverage with deductibles up to 20% of annual income, then patient pays 20% co-insurance.
New Brunswick	No restrictions	Voluntary coverage with premiums, then co-payments of $5 to $30 per eligible prescription, depending on income.
Prince Edward Island	No restrictions	Catastrophic coverage with deductibles up to 12% of annual income, then full coverage of eligible drugs.

Source [93]: Authors summary of information in CIHI (1028) National Prescription Drug Utilization Information System Plan Information Document, July 2018 supplemented by information from public drug plan websites, where necessary

Two provinces, Alberta and New Brunswick, offer the general population the option to purchase premium-based public drug coverage, which is subject to co-insurance on prescriptions filled. One province, Quebec, requires that all residents not eligible for private insurance, by way of their occupation, to purchase premium-based public drug coverage, which is subject to monthly deductibles and co-insurance on prescriptions filled.

Many Canadians have private insurance for prescription drugs. In Quebec, private insurance has been mandatory since 1997 for employees who qualify for extended health benefits as part of their compensation packages [15]. In all other provinces, private insurance is available on a voluntary basis and generally obtained only through extended health benefits as part of compensation packages negotiated between employers and unions. It is estimated that approximately two-thirds of Canadian workers have private insurance coverage [16]. Employees who work full-time, earn over $30,000, and over the age of 25 are more likely to have access to such private insurance coverage than part-time workers, those earning lower wages, and those under age 25 [16]. In 2016, 59% of Canadians reported having some form of private drug coverage [17]. Deductibles in private insurance plans are rare, applying to plans for just 11% of privately insured Canadians; however, most citizens who have private drug insurance pay co-insurance (67% of all beneficiaries) or fixed co-payments (17% of beneficiaries) [18].

Overall the “patchwork” system of private and public drug coverage in Canada leaves approximately one in five Canadians reporting that they have no coverage for their prescriptions [17]. Several surveys conducted over the past 15 years have found that approximately one in ten Canadian patients do not fill prescriptions written for them as a consequence of out-of-pocket costs [17, 19–22]. International comparisons have shown that, although access to medicines is higher in Canada than the United States, Canadians experience higher rates of cost-related non-adherence to medications (10.2%) than do residents in comparable high-income countries with universal drug coverage (average of 3.7%) [23]. This is estimated to result in hundreds of premature deaths annually in Canada, relative to the health outcomes that would be achieved if Canada had the same rates of cost-related non-adherence to medications as is found in comparable countries with universal drug coverage [24].

## Pharmacare economic considerations

Being informed on the economics of universal pharmacare requires, first and foremost, an overview of current prescription drug expenditure trends in Canada. Following this, an analysis of previous pharmacare simulations and their associated fiscal projections will provide an important summary when looking to the future on this topic.

### Current prescription drug expenditures in Canada

Canadians spent approximately C$33.9 billion on prescription pharmaceuticals in 2017, or C$926 per capita [25]. Prescription drugs accounted for 14.0% total healthcare spending in Canada in 2017, the third largest category of expenditure after hospital costs at 28.3% and physician services at 15.4% [25]. Over the past 30 years, total prescription drug expenditure in Canada has grown at an average annual rate of 8.1% while physician and hospital expenditures have trailed behind at average annual increases of 5.6% and 4.4% respectively [25]. And while growth of pharmaceutical expenditure slowed from 2011 to 2014, prescription drug costs still outpace the cost of physicians’ services and hospital care: prescription drug costs grew by 5.5% in 2017 while physician and hospital costs grew 4.4% and 2.9% respectively [25].

Part of the reason pharmaceutical costs have outgrown other major health expenditures in Canada stems from global changes in the availability and price of prescription drugs since the 1980s [26]. Another contributing factor is the difference in its system of financing medicines versus how it financed medical and hospital care. Canada’s system of universal, comprehensive, public insurance for medical and hospital care publicly finances 98% of all expenditures on physician services and 90% of all expenditures on hospital care [25]. In contrast, only 42% of total prescription drug expenditures are financed by public programs in Canada [25]. The balance of prescription expenditures in Canada are financed by private insurance plans (35%) and by out-of-pocket payment by patients (23%).

Provincial drug plans apply a variety of tools to control the expenditures under their programs. All provinces apply generic substitution policies or generic reference-based reimbursement policy, and a few provinces apply therapeutic reference-based reimbursement policies [27]. For example, in regards to therapeutic reference based compensation, the public plan may pay the cost of the lowest-cost Angiotensin Converting Enzyme inhibitor and allow patients to pay additional costs if they would prefer another molecule. Over the past decade, provinces have also increasingly used confidential rebate negotiations to obtain better prices for patented drugs than the manufacturer would allow them to obtain in a transparent fashion [28].

In 2010, Canada’s provinces and territories began to jointly negotiate brand-name prices and jointly set terms for generic drug pricing through the pan-Canadian Pharmaceutical Alliance [29, 30]. These negotiations set out the pricing terms in a mutually agreed upon letter of intent between governments and a drug’s manufacturer. Because final decisions regarding drug coverage reside in the individual provinces, manufacturers are not necessarily guaranteed coverage under all public drug plans in Canada even if pricing terms are agreed upon by the pan-Canadian Pharmaceutical Alliance [31]. This, combined with the fact that provincial drug plans finance less than half of all expenditures on prescriptions in Canada limits the power and impact of these negotiation processes.

As such, it is partially the uncoordinated mix of prescription drug financing that has resulted in less favorable conditions for controlling drug spending than are found in other high-income countries with universal drug coverage [32]. In 2015, for example, total per capita expenditure on pharmaceuticals in Canada was 43% higher than the average for the Organization for Economic Cooperation and Development (OECD) countries, surpassed only by the multi-payer systems of the United States and Switzerland [33]. Despite that Canada has a slightly younger population than some comparable countries (such as France, the Netherlands, Sweden, and the United Kingdom) [34], it spends more on medications per capita than these nations [35]. Research indicates that more coordinated systems of drug financing, particularly those that consolidate purchasing power in price negotiations with drug manufacturers, achieve lower prescription drug expenditures through lower prices and more cost-conscious prescribing patterns than Canada achieves [36, 37].

### Simulated pharmacare models

A number of recent studies have attempted to quantify the potential savings that a single-payer pharmacare program would provide Canada [38–43]. Published studies have indicated that a single-payer system could generate between $4 billion and $11 billion in annual savings for Canada [42–44]. These simulated results prompted Canada’s parliamentary Standing Committee on Health to commission the Parliamentary Budget Officer of Canada to produce government estimates of such a program [18]. In 2017, the Parliamentary Budget Officer estimated net annual cost reductions of $4.2 billion annually, or approximately 17% [18]. Importantly, these estimated savings also take into account the prospect of increased utilisation among those who currently lack coverage. The Parliamentary Budget Officer, for example, estimated that there would be more than 50 million additional prescriptions filled in Canada under a universal pharmacare program [18].

A number of proposals arguing for the enactment of particular policy frameworks for universal pharmacare, put forward by various sources in recent years, have enabled some political traction on the issue [38–43]. Five of the most recent pharmacare simulation models, with their associated cost reduction estimates, are briefly summarised in Table 2.

**Table 2:** – Pan-Canadian Pharmacare Models and Associated Fiscal Projections

	Data Sources	Type of Coverage	Model Assumptions	Financing Source	Net Difference in Total System Expenditures
Morgan et al. (2015) [40]	-Canada Rx Atlas 2012/2013 for drug utilisation data [94] -International drug pricing data from PMPRB	-Public Universal ‘First Dollar’	-Competitive pricing estimates as per singe-payer bulk-purchasing arrangement	-Federal/Provincial/Territorial (F/P/T) Government funding (tax based) -Small patient co-payments based on tiered national formulary	-Annual expense reduction of $4.2 to $9.4 billion
Gagnon and Canadian Federation of Nurses Unions (2014) [43]^a^	-Canada Rx Atlas for drug utilisation data -International drug pricing data from PMPRB	-Public Universal ‘First-Dollar’	-Competitive generic pricing estimates as per singe-payer bulk-purchasing arrangement −10% increase in base-expenditures due to higher utilisation -Improved evidence-based formulary management −2% reduction in dispensing fees -PMPRB pricing reform	-F/P/T Government (tax-based) -Small patient co-payments based on tiered national formulary	-Annual expense reduction of $2.7 to $11.5 billion
PDCI Market Access inc. (2016) [41]^a^	-Canadian Drug Claims Database -International pricing data from PMPRB	-Varies ranging from fully public to fully private	-Numerous assumptions dependent upon model	-F/P/T Government funding (tax-based) -Private plan premiums -Patient co-payments	-$1.92 billion in annual expense reductions or up to $350 million in increased annual expenses
Canadian Centre for Policy Alternatives & Canadian Doctors for Medicare (2017) [38]^a^	Canadian Institute for Health Information (CIHI) and previous studies	-Public Universal ‘First-Dollar’	-Unclear -Data derived from various sources for approximate calculation	-F/P/T Government funding (tax-based)	-approximately $30 billion in total savings (unspecified time period)
Parliamentary Budget Officer (2017) [18]	-CIHI datasets -Quintile IMS datasets	-Public Universal ‘First-Dollar’	-Competitive generic pricing estimates as per singe-payer bulk-purchasing arrangement -Quebec drug formulary	-F/P/T Government funding (tax-based) -$5 co-payments (on patented products)	-annual expense reduction: $4.2 billion

^a^Commissioned report from external stakeholder

Gagnon et al. made the economic case for universal pharmacare in 2010 which was later updated in 2014 [42, 43]. He asserts that a universal ‘first-dollar’ model would save between $2.7 and $11.5 billion annually, dependent upon whether favorable pricing reforms were implemented in tandem by the Patented Medicine Prices Review Board (PMPRB) [43]. Morgan et al., in a 2015 simulated cost study found that, even in their “worst-case” scenario model, $4.2 billion would be saved overall on prescription drug spending with implementation of universal ‘first-dollar’ pharmacare [44]. A 2017 study comparing 10 developed nations lend further support to these findings, showing that countries utilising single-payer models with evidence-based drug coverage criteria had lower average drug expenditures over countries with multiple payers [36]. The most recent iteration of a universal pharmacare proposal from Morgan and Gagnon et al. is their pharmacare 2020 proposal, which, though remaining otherwise consistent with previous publications, recommends federal assistance to each province in the form of an additional funding transfer to cover 25% of the added public expenses for universal pharmacare [45].

Contending the findings of Morgan et al., PDCI Market access inc. released a report, commissioned by the Canadian Pharmacists Association, which claimed that “existing proposals for national, exclusively publicly funded, pharmacare programs are impractical, disruptive, and overstated in regards to cost-savings”^42(pg 4)^ Instead, their analysis consisted of a number of models based on differing co-pay structures and population coverage (funding only the uninsured or all Canadians) [41]. Models that varied the patient co-payment, based on a publicly funded first-dollar universal model, all produced the same modest estimate of ~$1 billion in cost-savings. This represented a reduction in savings of over $3 billion from Morgan et al.’s ‘worst-case’ model. PCDI instead favor an approach where coverage is expanded for the under-insured population who currently lack equitable drug access, while maintaining the public-private insurer mix. This drug coverage strategy supposedly offers net cost-differences from current baseline spending ranging from a ~$2 billion surplus to a $350 million spending deficit, depending on the model [41]. However, given the commissioned nature of this report and its seemingly unverifiable methodology, it is best understood in the broader political context as informed by external stakeholder interests.

Perhaps the most definitive report so far on the costs and economic outlook of pan-Canadian pharmacare comes from the parliamentary budget officer of Canada [18]. The parliamentary budget office estimated net annual cost reductions of 17%, amounting to projected savings of ~$4.2 billion annually [18]. This was an important finding from government because it is largely in accordance with previous estimates from the academic community involved in health economic policy research (i.e Morgan, Gagnon et al.). Furthermore, the transparent methodology and reporting was accompanied by an exploration of various assumptions through sensitivity analysis. Although, the sensitivity analysis demonstrated the volatility of estimates under varying model assumptions.

Combined with the gaps in access to medicines within and across Canada, the evidence of poor cost-control under Canada’s patchwork system of private and public drug coverage has been a key motivation behind the recommendations by various government commissions that Canada adopt a universal system of pharmacare – including the 2018 recommendation of the Standing Committee on Health that the government act on implementing a pharmacare model consistent with the one costed by the Parliamentary Budget Officer [9].

## Pharmacare policy considerations

As Canada considers implementing some form of universal prescription drug coverage, there are several logical issues to consider. Among the most important policy questions are which drugs would be covered, who will be covered, what level of coverage will be offered, and who will administer the program?

### What drugs will be covered?

A system of universal drug coverage for Canada requires, first and foremost, the determination of the medicines that every Canadian will have coverage for. This will require a national formulary, a common list of eligible drugs for coverage, with or without additional drug-specific coverage criteria, that defines the minimum benefits that all Canadians would be entitled to. There have been several studies of existing public drug plan formularies in Canada, the most recent of which has demonstrated that there already exists an “implicit” national formulary by way of the extensive overlap of drugs listed in major therapeutic categories [46, 47]. The areas where there is already agreement among the provincial public drug plans in Canada may become the starting point for a national formulary under a universal pharmacare program.

A national formulary might, alternatively, approximate an “essential medicines” list for Canada. Essential medicines lists, such as the model list of the World Health Organization, comprise drugs selected as “…those that satisfy the priority healthcare needs of a population.” [48] Canadian clinicians and researchers have created a Canadian essential medicine list that comprises 125 medications that suit the majority of prescribing needs in Canada’s primary health care system [49]. Economic analysis suggest that universal coverage of that list would be a small but pragmatic step toward more comprehensive universal pharmacare, one that could generate $4.27 billion in annual savings for patients and private drug plan sponsors, at an incremental government cost of $1.23 billion per year [50].

An important distinction in the discussion of a national drug formulary is whether the concept implies a “minimum” standard for coverage within each of Canada’s provinces and territories. This idea of a minimum standard has been mentioned in some policy documents, including the 2018 report of the Standing Committee on Health [9]. In this context, “minimum” implies that provinces and territories can retain partial autonomy by having the freedom to go beyond the required drug listing, according to their own assessment of factors within their respective jurisdictions. In other words, while provincial/territorial drug program administrators would not be able to de-list drugs from the national formulary within their jurisdiction, they would be permitted to add drugs at their own discretion. If the national formulary was only to comprise an essential medicines list, it would be likely that most provinces would add to it. However, it is possible that a comprehensive national formulary would include more drugs than some provinces currently provide coverage for, making coverage beyond that list unlikely.

Whether the national formulary is to be comprehensive or narrow, it would likely be created by way of modifications of existing health technology assessment processes at the Canadian Agency for Drugs and Technologies in Health and the price negotiation processes of the pan-Canadian Pharmaceutical Alliance. The former agency provides formulary listing recommendations to provincial members of the latter, in the form of clinical and cost-effectiveness assessments, via their Common Drug Review and pan-Canadian Oncology Drug Review services [51, 52]. However, currently the individual provincial policymakers are under no obligation to accept the recommendations coming from reviews of the Canadian Agency for Drugs and Technologies in Health. A recent high-level government advisory committee has recommended that the roles and responsibilities of such pan-Canadian agencies be aligned with the vision of creating and maintaining an evidence-based national formulary for a universal pharmacare program for Canada [53]. This would not necessarily increase public administration costs nor necessarily preclude agencies from doing some technology assessment and price negotiations for consideration on a case-by-case and province-by-province basis.

### Who will be covered?

The concept of “universal” pharmacare self-evidently suggests that all Canadian citizens and permanent residents be granted the same treatment under any future coverage framework. Thus, the precedent that has long been established by the *Canada Health Act* is probably the best indicator for predicting who would be covered. However, the question remains as to how the policy framework would be resolved to address the status of other persons residing in Canada such as refugees, permanent residency applicants, extended travellers and undocumented persons.

As alluded to in the case of permanent residents, the Government of Canada has previously entitled this group of persons to the same level of universal “medically necessary” coverage as Canadian citizens [54]. As such, it would be unexpected for any future pharmacare plan to exclude permanent residents as this would be an unequal extension of the already existent universal coverage. Likewise, for those not meeting permanent resident status, the benefits that fall temporarily under the Interim Federal Health Program (which are very similar to coverage for citizens) may be extended and re-organized to reflect any new, universal pharmacare program [55]. Regardless of whether prescription drug coverage changes for this group, the separation of the Interim Federal Health Program from the conventional “medicare” system covering citizens will likely remain, if only for epidemiological government data collection purposes.

### What level of coverage will be offered?

Canadian Medicare is a ‘first-dollar’ health insurance program. Essentially, the healthcare provider or institution bills the province/territory for the cost of goods and services rendered, at no direct cost to the patient. This is in accordance with the principles of the *Canada Health Act*, which provinces are obliged to adhere to if they wish to receive federal government funding in the form of the federal health transfer [56]. Several commissions and government reports have recommended that a national formulary of medicines be added to the *Canada Health Act*, which would appear to imply that similar prohibitions on prescription user-charges would apply [6–9]. This would make Canada not unlike that of the United Kingdom nations; Wales, Scotland and Northern Ireland (but not England) where prescription charges were incrementally abolished by the National Health Service throughout the 2000’s [57]. But such a program would be a radical transformation of existing public drug plans in Canada, virtually all of which employ deductibles, co-insurance, and/or fixed co-payments for eligible beneficiaries.

At the other extreme in terms of benefit designs under universal pharmacare would be to set the national standard at the level of catastrophic coverage against high costs. Given that such ‘last-dollar’ coverage is commonplace in the provinces, designing universal pharmacare in Canada around this option would be a national move to establish uniform catastrophic thresholds. This may result in an increase or decrease in what patients already must pay depending on their province of residence and the agreed upon national threshold. Furthermore, nationalizing catastrophic coverage as the standard of public drug benefits in Canada may not address the goals of improving access to medicines since the province wherein such a ‘last-dollar’ coverage model is applied universally (British Columbia) experiences the highest rates of cost-related non-adherence to medications in Canada [17].

Last-dollar coverage is, however, appealing to industry interests, which could reduce the political costs of program implementation. Drug manufacturers prefer this model of coverage because it makes the government plan a payer of last resort only, rather than the single-payer for covered drugs. Under those circumstances, a majority of drug purchases by a majority of patients will be below deductible, meaning the patients will have to pay for them whether they are on the national formulary or not. This diminishes the negotiating power of the public plan because it reduces the impact on demand of a positive formulary listing [58]. Private insurers also prefer a ‘last-dollar’ model for universal public pharmacare because it leaves considerable room for them to sell private insurance for costs not covered by the public plan. This is because private insurance generally will offer some level of immediate and perceived financial benefit to the enrollee which is otherwise not realised for many patients under ‘last-dollar’ public coverage alone. Additionally, a ‘last-dollar’ model of pharmacare leaves private insurers with a market for covering routine operating expenses while eliminating their exposure to the highest cost medicines (i.e., the catastrophic expenses that would be incurred by government).

### Who will administer the plan?

A final consideration is who will administer universal pharmacare? There are two dimensions to this question in the Canadian federation. First, will the program be run as a public program or will private insurers be providers of the pharmacare plan? Second, will the public portion of the program be provincially or federally administered?

If universal pharmacare in Canada is to be a catastrophic, ‘last-dollar’ model of public drug coverage, then there will remain a major role for private insurers to provide plans for those interested and able to cover expenses below their annual deductibles and for medicines not on the national formulary. This is a form of complementary private insurance wherein the core, universal benefit of protection against catastrophic drug costs would remain a public responsibility. Because most provinces already offer catastrophic drug coverage for most or all of their populations, this would imply little or no change in the role of the private insurance sector in Canada’s system. Administration costs would therefore likely remain as they are in both the private and public sector; higher in the private than public sphere [59]. Also, purchasing power would likely remain fragmented and therefore limited by comparison to a single-payer system.

If universal pharmacare in Canada is to be a more comprehensive benefit for medicines on the national formulary, it is possible that such a program could be a single-payer public plan for such medications or a mandatory private plan for such medications. Universal drug coverage can be achieved in a manner like Quebec has had since 1997. In their system, private insurers are the primary providers of drug benefits in the sense that all employees who qualify for private insurance for prescription drugs as part of their compensation packages must have such private insurance. This program limited the public sector cost of implementing universal drug coverage; however, it also limited both incentives and capacity for cost control, which has resulted in far higher prescription drug costs in Quebec than in the rest of Canada [15].

If pharmacare is to be implemented as a single-payer system for drugs on a national formulary, such a system will have significant purchasing power and superior administrative efficiency [38, 39, 59]. It also has the benefit of achieving greater equity and efficiency in revenue collection, which can come from changes to existing sources of government general revenues (such as incremental increases in personal and corporate income taxes). These are among the reasons that a single-payer model has been recommended by major commissions and government committees. Under such circumstances, private insurance would likely remain for individuals who wished to have choices beyond the drugs listed on the national formulary, and possibly for those who wished to have pre-payment plans for the co-payments or co-insurance that the universal public benefit might still have.

Even if there is to be a single-payer, public model of pharmacare for the drugs on a national formulary, a final question would remain: would the program be run by provinces or by the federal government. Because the *Canadian Constitution Act*, in conjunction with the *Canada Health Act*, assigns provinces primary responsibility for matters related to health care administration, it is likely that universal pharmacare would be run by individual provinces but held to national standards established by federal legislation. This legislation would define the terms by which it would grant funding to support the pharmacare program(s). As an aside, how the pharmacare framework is legislatively enacted, be it through changes to the existing *Canada Health Act* or by the drafting of a new statute is an important topic, but one that goes beyond the scope of our review. For the interested reader, this legislative issue has recently been taken up in detail elsewhere [60].

Regardless of the legislative operationalization of the policy framework, universal pharmacare, under the aforementioned assumptions, would be tantamount to how Canadian medicare is run and could be a viable option if the federal government was willing to put sufficient funding into the system. As the provinces currently spend over $12 billion per year on their existing public drug plans, it is likely that the federal government would have to come up with most, if not all, of the incremental public expenditure needed to make those programs compliant with a national standard for universal pharmacare. Based on the estimates of the Parliamentary Budget Officer, that would require $7 billion or more in federal funding – though it is worth noting that the Parliamentary Budget Officer estimates that the net benefit to taxpayers would be a $4 billion savings [18].

## Pharmacare political considerations

The federal government’s recent creation of an Advisory Council on the Implementation of National Pharmacare in 2018 suggests that some form of pharmacare may be forthcoming, likely after the 2019 election campaign in which promises of universal pharmacare may be a differentiating factor between conservative and progressive political parties [12]. Political considerations are therefore paramount at this point in the policy development cycle. To what extent is the promise of a universal pharmacare program something that will help the current federal government get re-elected or an opposition party elected? As this is a major health care issue, an important political consideration is the support or opposition from health professional groups, health charities, industry and patient organizations.

### Health professionals

Among professionals, physicians and nurses have become increasingly vocal advocates for a universal, comprehensive pharmacare program. The most vocal physicians have been the members of Canadian Doctors for Medicare, an organization that has actively campaigned for universal pharmacare on the grounds that such a program would increase access to medicines, improve patient outcomes, reduce administrative burden on physicians, and save money through bulk purchasing [38, 61]. The Canadian Medical Association has taken a more tentative stance, however, arguing that universal coverage is necessary but that it need not be comprehensive nor fully funded through public financing [62].

Nurses have also been active in advocating for national pharmacare [43]. Since 1991, the Canadian Federation of Nurses Unions have campaigned for universal pharmacare through a variety of organizational activities [39, 43, 63, 64]. Their campaigning is based on similar logic as that of the Canadian Doctors for Medicare; however, the nurses unions enjoy the support of other nursing organizations, such as the Registered Nurses Association of Ontario, who have added their voice to the call for universal, comprehensive, public pharmacare [65].

On behalf of the pharmacy profession, the Canadian Pharmacists Association has been the leading voice in the pharmacare dialogue. They have regularly updated their views and recommendations on this issue over the years as the policy discourse has developed [66–70]. In the past, they have commissioned an external report on pharmacare costing to assist in informing their economic position [41]. Overall, they have been more conservative than other health profession groups; cautioning government about the potential for health system efficiency issues that may arise from universal ‘first-dollar’ coverage implementation such as drug shortages, interrupted access to medicines no longer covered, and potentially inappropriate drug prescribing choices [67]. However, the Canadian Pharmacists Association has consistently advocated for reinvestment of cost-savings from potential lost dispensing fee revenue back into pharmacy business in the form of funding for clinical pharmacy services; an argument based on the cost-effectiveness of improved health outcomes resulting from pharmacist intervention [67, 69].

The conservative position of the Canadian Pharmacists Association may be reflective of the significant and precarious state imposed on pharmacy business from pharmaceutical market reform (increasing drug genericization and provincial policy responses) that have resulted in reduced revenue from dispensing fees and product mark-ups. How much influence large, corporate, chain-drug stores have had on forming the Canadian Pharmacists Association’s position (directly or indirectly) is debatable but worthy of consideration. This speculation is based on past observation of opposition by corporate chain-pharmacy to Quebec’s proposed public prescription drug funding which occurred in the 1990’s [15, 71]. Importantly, as payment mechanisms have always been dependent on provision of pharmaceuticals as the core service, there has been a somewhat silent struggle by pharmacists, largely unbeknownst to the public and other health professions, to rectify the smaller patchwork of clinical pharmacy service remuneration simultaneously with the drug coverage patchwork [72]. Nonetheless, this revenue-based concern is largely exempt for hospital pharmacists, who are not reliant on dispensing fees for their income. Perhaps this explains why their representative organization, the Canadian Society for Hospital Pharmacists, has advocated instead for a universal pharmacare framework that appears closer to that of other healthcare groups in the nursing and medical professions [73].

### Public opinion and representation

A groundswell of major national public interest groups, consisting of diverse representation ranging from charities, advocacy organizations and academia to labour unions and retiree associations, have added their voices to the pharmacare debate in recent years. Among these groups, the Health Charities Coalition of Canada, the Canada Health Coalition, Canada Labour Congress, the Canadian Association for Retired Persons and ‘Pharmacare 2020’ (health affiliated research professors) have come out in support of universal pharmacare [45, 74–79]. Nevertheless, the extent of their involvement and the content of their policy prescriptions have varied considerably. Beyond organized advocacy, polling data and focus groups such as the Citizens’ Reference Panel on Pharmacare have backed the need for implementation of universal pharmacare [80, 81].

Other patient advocacy groups, such as the Canadian Organization for Rare Disorders, while in support of universal pharmacare in principle, have raised concerns about access limitations to expensive, rarely used agents that may ensue after program implementation [82]. These concerns stem justifiably from fear that a restricted minimum national formulary, nested in a single-payer model, may radically disrupt pre-existing financial support arrangements in the form of private payer insurance.

In spite of the widespread support for universal pharmacare, there remains staunch opposition from some conservative political think tanks such as the Fraser Institute and the Taxpayers Federation [83, 84]. Essentially at odds with the socialist principles underlying a universal ‘first-dollar’ pharmacare model, their foremost considerations are limiting the financial burden to the taxpayer while preserving the market for private competition.

### Industry

The interests of the Canadian pharmaceutical industry have been prominently represented by Innovative Medicines Canada, who represent more than 45 drug manufacturer members [85]. In attempts to remain ‘politically correct’ in their positions, they have been openly supportive of a national pharmacare strategy, albeit with the important caveat that any decided solution not limit the current insurable coverage situation of any Canadian [86, 87]. The motivating interest underlying this position is to maintain the ongoing operation of private insurance company formularies, which often cover patented drug products that are not eligible benefits under public plans. And so, a switch to a universal ‘first-dollar’ public plan, based on rigorous cost-effectiveness assessment, would potentially exclude expensive industry products where sales figures were previously disproportionately reliant on private plan coverage.

The Canadian Life and Health Insurance Association, as the voice of private insurers, has been diligent in issuing press releases and responses to various reports and news developments related to universal pharmacare [88–92]. Echoing that of the pharmaceutical manufacturers, the private insurance industry position has strongly maintained that a universal pharmacare plan should co-exist with private third parties and not threaten the holdings of beneficiaries of private insurance [92]. To this end, they have invoked the existence of their ~ 26 million beneficiaries, who they claim generally receive broader and more extensive coverage than public plans, to imply the potential entailment of negative consequences on the Canadian public from a radical change to existing coverage arrangements. Essentially, they contend that, should the federal government implement single-payer, ‘first-dollar’, universal coverage, an estimated $14–20 billion would need to be immediately earmarked for public drug expenditure that was previously reimbursed privately [88, 91].

## Conclusion

After more than 60 years of recommendations from national commissions and government committees, Canada appears poised to implement some form of universal pharmacare, likely in the government mandate that would start after the federal election in late 2019. Many stakeholder groups, academic experts, and government committees have more or less agreed on many of the major framework characteristics. Namely, universal pharmacare in Canada would, ideally, involve a reasonably comprehensive, evidence-based national formulary that is covered by a single-payer public program involving limited direct charges to patients. Private insurance would be a voluntary and complementary option for covering additional drug choices and prepaying any user charges that the universal public system may entail.

But the policy process isn’t over until it is over. The concept of a universal, single-payer pharmacare program in Canada looms as a significant threat to the interests of industry; private insurers and drug manufacturers who stand to lose the most from the program. There is also opposition from citizens concerned about tax increases, even if such increases produce net savings to taxpayers, and those who simply do not wish to have the role of government expanded within Canada’s health care system.

Whether Canada moves forward on reforms will therefore depend on voter mobilization. This will be influenced, in part, by the work of the Advisory Council on the Implementation of National Pharmacare. If the panel develops a proposal that resonates with government and, importantly, the public, it may become a roadmap and touchstone in public debate and form the basis of the current government’s 2019 election platform. However, policy development will also depend on the balance of political power of groups involved in the sector. The stakeholders who stand to lose revenues and profits under a universal pharmacare program have more concentrated interests than those who stand to gain; as such, it is possible that those opposed to major policy reforms may be able to launch marketing campaigns that change the narrative in ways that may make it difficult for reforms to take place. Time will tell.

## Additional file

Additional file 1: Overview of Major Entities in Canada’s Pharmaceutical Market Environment.( PDF168 kb)

## Footnotes

1 We have excluded Canada’s territories from table 1 because of the greater reliance on federal drug program funding in the territories and their relatively small share of the Canadian populations (the three territories combined account for less than 1% of Canada’s total population).
