# Supplementary material for: Prescription drug coverage in Canada: a review of the economic, policy and political considerations for universal pharmacare
Source: J Pharm Policy Pract. 2018 Nov 7;11:28. doi: 10.1186/s40545-018-0154-x (PMC6220568; doi:10.1186/s40545-018-0154-x)
Supplement: Supplementary file 1 — Overview of Major Entities in Canada’s Pharmaceutical Market Environment.( PDF168 kb) [file 40545_2018_154_MOESM1_ESM.pdf]

Appendix 1 – Overview of Major Entities Operating in Canada's Pharmaceutical Market Environment

Health Canada.

The role of prescription and non-prescription drug regulation within Canada falls to Health Canada's 'Health Products and Food Branch's 'Therapeutics Products Directorate' and 'Natural and Non-Prescription Health Products Directorate'.[1,2] These directorates are divided into numerous bureaus with various scientific and evaluative functions to ensure that new products, submitted for marketing authorization, meet evidentiary standards relating to safety, efficacy, quality, labeling and packaging.[1,2] After a new prescription drug product has undergone review, and if all criteria are sufficiently met, a Notice of Compliance (NOC) is issued to the manufacturer which serves as both a marketing license and a statement of the required post-marketing activities that must be undertaken to monitor for safety and quality control issues.[3]

Canadian Agency for Drugs and Technology in Health

The Canadian Agency for Drugs and Technology in Health (CADTH) is an independent, not-for-profit, health technology assessment agency that conducts evidence-based and cost-effectiveness reviews on drug products and medical technologies for the Canadian healthcare system.[4] The Common Drug Review (CDR) and the pan-Canadian Oncology Drug Review (p-CODR) are CADTH programs targeted at provincial policy makers to guide their drug formulary listing decisions. Despite that CADTH has no formal decision-making power in respect to coverage or pricing decisions on drugs, they nevertheless have significant influence among policymakers of public and private drug programs.

The Patented Medicine Prices Review Board

In 1987, the Patented Medicine Prices Review Board (PMPRB) was founded as a federal, quasi-judicial, consumer protection agency with the mandate of ensuring patented medicines in Canada are not "excessive" in their market pricing relative to the consumer price index and the costs in seven nations of comparative economic development; France, Germany, Italy, Sweden,

Switzerland, the United Kingdom and the United States.[5] Since then, their role has evolved to encompass broader reporting on drug utilisation trends for public drug programs by leveraging the data collected from the National Prescription Drug Utilisation Information System (NPDUIS).[5–8]

Most importantly, the PMPRB operates by monitoring pricing and communicating directly with industry representatives when the market cost for a patented medicine is deemed inappropriately high. This role essentially provides the check and balance against the temporary monopolistic power of industry patent-holders, especially in the case of a medical breakthrough drug with no competitors. However, compliance with board recommendations on revised pricing is only enforceable after legal proceedings. The extent to which the PMPRB has faithfully executed their duty as a regulator, in keeping with the public interest, has been questioned.[9,10] One major criticism is that the international standard of comparison (the 7 aforementioned nations) establishes a standard that is unfairly high for reference pricing into the Canadian prescription drug market.[9–11] In addition to this, marketing before patenting has been detected as a jurisdictional loophole that has enabled industry, in certain cases, to market medicine at unreasonable prices for extended periods after receiving their NOC but before filing for patent protection.[12]

Pan-Canadian Pharmaceutical Alliance

The pan-Canadian Pharmaceutical Alliance (p-CPA), founded through Canada's premiers (Council of the Federation) in 2010, is comprised of drug policy decision-makers from each province/territory.[13,14] The purpose of the p-CPA is primarily that of collective negotiation with drug manufacturers, with the goal of establishing reasonably equitable and consistent prices for new drugs across jurisdictions. The p-CPA has routinely found itself involved in negotiations with industry representatives, regarding single drug products, to draft a mutually agreed upon letter of intent. Expected adherence to the letter of intent would take the form as official product listing agreements at the prior negotiated price, in each respective jurisdiction.

Where the PMPRB regulates "ceiling prices" on brand name drug products, the p-CPA negotiates down these prices to the advantage of government, wholesalers, pharmacies and ultimately, patients. Recently however, the p-CPA has proven the magnitude and breadth of their negotiating power beyond patented products via the establishment of a five-year plan with the Canadian Generics Pharmaceutical Association (CGPA) wherein 70 commonly used generic drugs

saw price reductions in the range of 25-40% each.[15,16] This has already been lauded a major national success with an anticipated $3 billion being saved over the next 5 years.[16]

Pharmaceutical Industry

The pharmaceutical industry (i.e drug manufacturers) are represented nationally by Innovative Medicines Canada (IMC).[17] Members of IMC regularly interact formally with Health Canada, prior to market entry, regarding product licensing and quality assurance. Their members are also monitored and quasi-regulated by the PMPRB on medication costs. Their role in research and development of emerging therapeutic products on Canadian soil offers a stimulus to the domestic economy. Government has been incentivised to maintain mutually beneficial relationships with these companies to reap these benefits. To some degree, these relationships are necessarily maintained by ensuring favorable enough market conditions to achieve adequate profit margins for these companies via pharmaceutical sales.

The PMPRB reports that this was pursued in the past by enabling patent extensions, under the assumption that investment by industry in research and development would increase as a show of good faith.[5] However, research and development activity has continued to be underwhelming, eliciting cries from economic spectators that Canada's poor rate of return in research and development relative to the prices and market exclusivity provided to industry products is not reflective of international fairness.[18]

Private Health Insurance Companies.

Private health insurance companies contribute upwards of 40% of total payment towards prescription drugs.[19] The collective interests of private health insurers in Canada are represented through the Canadian Life and Health Insurance Association (CLHIA).[20] To some extent, CLHIA approximates the role of a privatised equivalent of the p-CPA. Unlike the p-CPA however, their role goes beyond drugs into the insurance of health services. They have been active in presenting their views on issues related to drug coverage and national Pharmacare.

References

1. Health Canada G of C. Natural and Non-prescription Health Products Directorate. https://www.canada.ca/en/health-canada/corporate/about-health-canada/branches-agencies/health-products-food-branch/natural-non-prescription-health-products-directorate.html. Published 2018. Accessed February 28, 2018.
2. Health Canada G of C. Therapeutic Products Directorate. https://www.canada.ca/en/health-canada/corporate/about-health-canada/branches-agencies/health-products-food-branch/therapeutic-products-directorate.html. Published 2018. Accessed February 28, 2018.
3. Health Canada G of C. Notice of Compliance - Drug Products. https://www.canada.ca/en/health-canada/services/drugs-health-products/drug-products/notice-compliance.html. Published 2018. Accessed February 28, 2018.
4. Canadian Agency for Drugs and Technology in Health. About CADTH. https://www.cadth.ca/about-cadth. Published 2018. Accessed March 11, 2018.
5. Patented Medicine Prices Review Board. *Strategic Plan : 2015-2018*. Ottawa, ON; 2015.
6. Patented Medicine Prices Review Board. *Generics 360: Generic Drugs in Canada, 2016*. Ottawa, ON: Patented Medicine Prices Review Board; 2018.
7. Patented Medicine Prices Review Board. *Alignment Among Public Formularies in Canada - Part 1: General Overview*. Ottawa, ON; 2017.
8. Patented Medicine Prices Review Board. *CompassRx: Annual Public Drug Plan Expenditure Report, 2015/16*. 2nd ed. Ottawa, ON: Patented Medicine Prices Review Board; 2017.
9. Zhang R, Martin D, Naylor D. Regulator or regulatory shield? The case for reforming Canada's Patented Medicine Prices Review Board. *CMAJ*. 2017;189(14):E515-516.
10. Skinner B. Canada's regulator of patented drug prices is rigging data, and risking our health. Financial Post. http://business.financialpost.com/opinion/canadas-regulator-of-patented-drug-prices-is-rigging-data-and-risking-our-health. Published 2017. Accessed February 28, 2018.
11. Morgan SG, Daw JR, Thomson PA. Pharmaceuticals. In: Marchildon GP, Di Matteo L, eds. *Bending the Cost Curve in Health Care: Canada's Provinces in International*

Perspective. 1st ed. University of Toronto Press; 2015:113-137.

12. Lexchin J. Marketing before patenting: implications for price controls in Canada. *Open Med*. 2010;4(3):139-142.
13. Husereau D, Dempster W, Blanchard A, Chambers J. Evolution of drug reimbursement in Canada: The Pan-Canadian pharmaceutical alliance for new drugs. *Value Heal*. 2014;17(8):888-894. doi:10.1016/j.jval.2014.08.2673.
14. IBM Canada Ltd. *Pan Canadian Drugs Negotiations Report*.; 2014. http://www.pmprovincesterritoires.ca/phocadownload/pcpa/pan_canadian_drugs_negotiations_report_march22_2014.pdf.
15. pan-Canadian Pharmaceutical Alliance, Canadian Generic Pharmaceutical Association. A Joint Statement from the pan-Canadian Pharmaceutical Alliance and the Canadian Generic. https://www.newswire.ca/news-releases/a-joint-statement-from-the-pan-canadian-pharmaceutical-alliance-and-the-canadian-generic-pharmaceutical-association-671651014.html. Published 2018. Accessed February 27, 2018.
16. The Canadian Press. Almost 70 commonly prescribed generic drugs to be discounted in Canada under new deal. Toronto Star. https://www.thestar.com/business/2018/01/29/almost-70-commonly-prescribed-generic-drugs-to-be-discounted-in-canada-under-new-deal.html. Published 2018. Accessed February 28, 2018.
17. Innovative Medicines Canada. About - Innovative Medicines Canada represents Canada’s innovative pharmaceutical industry. http://innovativemedicines.ca/about/. Accessed March 11, 2018.
18. Morgan SG, Gagnon MA, Mintzes B, Lexchin J. A Better Prescription: Advice for a National Strategy on Pharmaceutical Policy in Canada. *Healthc Policy*. 2016;12(1):18-36.
19. Candian Institute for Health Information. Drug Spending at a Glance. 2017:1-2.
20. Canada Health and Life Insurance Association. Mission and Objectives. https://www.clhia.ca/domino/html/clhia/CLHIA_LP4W_LND_Webstation.nsf/page/6EBFE54D9D076C568525780E0056B1BE?OpenDocument. Published 2018. Accessed March 11, 2018.
